# Association between Polymorphisms of the IL-23R Gene and Allergic Rhinitis in a Chinese Han Population

**DOI:** 10.1371/journal.pone.0063858

**Published:** 2013-05-16

**Authors:** Di Hu, Guohua Hu, Jing Zhu, Yang Shen, Houyong Kang, Suling Hong

**Affiliations:** Department of Otorhinolaryngology, the First Affiliated Hospital of Chongqing Medical University, Chongqing, People’s Republic of China; South Texas Veterans Health Care System and University Health Science Center San Antonio, United States of America

## Abstract

**Objective:**

Polymorphism of the interleukin-23 receptor gene corresponds with susceptibility to several immune-related diseases. For the terminal differentiation of IL-17-producing effector T-helper cells in vivo, the interleukin-23 receptor gene is of vital importance. As shown recently, Th17 cells probably have a great influence on the pathogenesis of allergic airway diseases. Our intention was to establish an association between polymorphisms in the IL-23R gene and allergic rhinitis (AR) in the Chinese Han population.

**Methods:**

We included 358 AR patients and 407 control Chinese subjects in a case-control comparison. The study involved obtaining blood samples for DNA extraction genotyping and determination of 4 selected single-nucleotide polymorphisms in IL-23R by performing PCR restriction fragment length polymorphism analysis (PCR-RFLP).

**Results:**

A substantially growing prevalence of the homozygous rs7517847 GG genotype and G allele appeared in the AR patients unlike that observed in the control individuals (P<0.001). In addition, substantially high frequencies of the GGCA and GGCG haplotypes were observed in the AR patients, unlike that observed in the control individuals (P<0.05). The results suggest that the AGTG haplotype may provide protection against AR (P<0.001).

**Conclusions:**

To the best of our knowledge, this is the first study to demonstrate an important association between polymorphisms in IL-23R and AR in the Chinese Han population. A strong association between rs7517847 in a SNP of IL-23R, and AR was identified.

## Introduction

In the past few decades, the prevalence of allergic diseases, including asthma, rhinitis, eczema, and food allergies, has risen sharply [1]. Allergic rhinitis (AR), which is a condition similar to common cold, could affect mental and learning capacities up to 30% (Global Allergy and Asthma European Network addresses the allergy and asthma epidemic) [2].In 4 major cities in western China, the prevalence of self-reported AR was 32.30%(Chongqing), 34.3%(Chengdu), 37.9%(Urumqi), and 30.3%(Nanning) [3]. AR is an inflammatory disease of the nasal mucosa induced by an immunoglobulin E (IgE)-mediated reaction in allergen-sensitized individuals. AR is a complex phenotype produced by the interaction of genes and the environment. It has been reported that numerous loci and candidate genes show an association with AR [4]–[8]. In spite of that, the molecular mechanisms underlying the development of AR have not been completely determined.

As shown recently, Th17 cells might have a great influence on the pathogenesis of allergic airway diseases. The IL-17 levels in the in nasal lavage, induced sputum, and serum of patients with AR showed a substantial increase [9]. In recent studies, IL-17A and IL-17F several single nucleotide polymorphisms (SNPs), and some intergenic variants were observed to be potentially associated with AR and comorbid asthma in the Chinese population [10]. For the terminal differentiation of IL-17-producing effector T-helper cells in vivo, the interleukin-23 receptor (IL-23R) gene, located on chromosome 1p31, is of vital importance, because its ligand, IL-23, is a major component of the immune regulatory pathway [11], [12]. As observed in recent studies, several SNPs in the IL-23R gene are found to be associated with immune-related diseases, including Crohn’s disease, rheumatoid arthritis, Behcet’s disease, and ankylosing spondylitis (AS)[12]–[14].

Therefore, in this study, we aimed to determine the association between IL-23R polymorphisms and susceptibility to AR. In accordance with our aim, we analyzed 4 SNPs, including rs17375018, rs7517847, rs1343151, and rs11209032.

## Methods

### Patients and Healthy Controls

From April to August 2012,358 patients aged 4 to 70 y were selected and evaluated on the basis of the ARIA criteria [15]. All the patients were of Chinese Han ethnic origin, and all were from the Chongqing city and its neighboring townships in the southwest region of China. All the patients were from and had been treated at the outpatient clinic of the Department of Otolaryngology Head and Neck Surgery at the First Affiliated Hospital of Chongqing Medical University, Chongqing, China. Factors such as medical history, patient complaints, nasal/endoscopic inspection, and the presence of positive allergen as per skin prick tests (SPT: Allergopharma, Hamburg, Germany), and responses to a panel of common allergens, were used to diagnose allergic rhinitis. A total of 18 inhaled allergens were tested, including house dust, grass, tree, mold, food, and weed panel allergens. The typical symptoms defined by ARIA include 2 or more AR symptoms (nasal congestion, rhinorrhoea, nasal itching, and sneezing) lasting for 4 or more days in a week in the year before the study was begun. A positive SPT result was defined as that where a wheal is larger than or equal to one half of the diameter of the histamine control, and at least 3 mm larger than the diameter of that shown by the negative control. Skin prick tests were performed by specialists, technicians, and nurses, while AR was diagnosed by the clinical rhinologists.

Meanwhile, 407 unrelated healthy subjects from the same region were enrolled as the control individuals during the same time. To evaluate the impact of occupation on the association of the 4 polymorphisms, we divided the samples into 2 groups according to the place of work (indoor versus outdoor work). All the control individuals were of the Han ethnicity of the general population and had undergone a comprehensive medical screening. They did not show clinical features or family history of allergies and had not experienced an upper respiratory tract infection in the 4 weeks prior to the study. Individuals diagnosed with AR and those with eczema, asthma, or a tumor in the nasal cavity were excluded from the study. The case-control ratio of our population (0.88∶1) was considered to be one of the parameters of analysis. The clinical characteristics of the AR patients were assessed at the time of diagnosis and are summarized in Table 1.

**Table 1:** Clinical features and demographic characteristics of the study population.

Characteristic	AR(n = 358)	Control(n = 407)
Gender[Male/Female]	188/170	200/207
Age[Mean(range)]	28.51(4–70)	34.09(7–75)
Occupation[indoor/outdoor]	200/158	230/177
Allergen Category[%]		
House dust mite	220(61.5)	–
Pollens	65(18.2)	–
Mixed allergens	73(20.4)	–

AR, allergic rhinitis.

### Ethics Statement

The local ethics authorities (the Ethics Committee of the First Affiliated Hospital of Chongqing Medical University, Chongqing, China) provided permission, and helped to obtain informed consent from all the participants. Written informed consent was obtained from all the participants. Informed consent was obtained from the next of kin, caretakers, or guardians of the minors and children participating in this study.

### SNP Selection and Genotyping

The SNPs were selected according to the results of the earlier studies on the association of IL-23R polymorphisms with other autoimmune diseases [11], [16]–[18]_ENREF_15_ENREF_15. Although rs17375018 was not shown to be associated with other diseases, it was found to be associated with Behcet’s in our laboratory of Ophthalmology GWAS (data not shown) and was, therefore, selected as one of the candidate SNPs investigated in the present study. We also selected rs11209032, rs7517847 and rs1343151 as candidate SNPs, as these SNPs had been demonstrated earlier by other groups to be associated with certain immune-related diseases [16], [19], [20].

All the blood samples were collected in ethylenediaminetetraacetate tubes and stored at −70°C until use. Using the QIAamp DNA Blood Mini Kit, genomic DNA was extracted and the target region was amplified by performing PCR. PCR restriction fragment length polymorphism analysis was applied to genotype these SNPs. The obtained digestion products were visualized on a 4% agarose gel and stained with Gold View (SBS Genentech, Beijing, China). In order to validate the method used in this study, Invitrogen Biotechnology Company (Guangzhou, Guangdong province, China) was assigned to execute direct sequencing by using randomly selected subjects (20% of all the blood samples).

### Statistical Analysis

Call rates were compared between AR and control by the χ^2^ test. The χ^2^ test was applied to compare the allele and genotype frequencies between the patients and control individuals.Estimation of genotype frequencies was performed by direct counting. The online software platform SHEsis (http://analysis2.bio-x.cn/myanalysis.php) was used to analyze the haplotype and probabilities. We used non-risk alleles as the reference, and tested all the other haplotypes. Logistic regression analysis was used to analyze the genotype allele, controlling for age, gender, and occupation as the covariables. All the statistical analyses were performed using the SPSS software (version 13.0).

## Results

Clinical and demographic characteristics of the patients and the control individuals are summarized in Table 1. To evaluate the impact of occupation on the association of the 4 polymorphisms, we divided the study participants into 2 groups according to their place of work (indoor *versus* outdoor). The groups were comparable with respect to mean age, gender, and workplace ratio. Pearson Chi-Square analysis of the ratios of male: female and indoor: outdoor work between the control and the study groups was performed (P>0.05). The mean age values for the control individuals and the AR cohorts were 28.51y (ranging from 4–70 y), and 34.09y (ranging from 7–75 y), respectively, which were not significantly different (P>0.05; measured by t-test). Further investigation was performed to determine whether any association existed between the IL-23R SNPs and certain allergens of AR, such as seasonal grass pollens, house dust mites, dog and cat fur, molds, and cockroach. Any association of these parameters with the tested IL-23R SNPs has not yet been determined. The 4 SNPs analyzed in the IL-23R gene were in Hardy–Weinberg equilibrium in both the AR cohort and the control cohort. The 4 SNP call rates in the patients and the control individuals and their HWE p-values are shown in Table 2. There was no different concerning the call rates between the AR group and the control (P>0.05).The genotype frequencies were obtained by direct counting of the genotyping results. The 4 SNPs of IL-23R (rs11209032, rs17375018, rs1343151, and rs7517847) were successfully genotyped for 382 AR patients and control individuals. The minimum SNP call rate was 92.7%.

**Table 2:** The 4 SNP call rates in patients and control individuals and HWE p-values.

SNP	Call rate (%)		HWE p-value	
	AR	Control	AR	Control
rs17375018	92.7%	92.0%	1.00	0.21
rs7517847	97.5%	95.0%	0.30	0.92
rs1343151	98.5%	98.0%	0.71	0.44
rs11209032	95.8%	96.2%	0.13	0.21

AR, allergic rhinitis, SNP; single-nucleotide polymorphism; HWE,Hardy-Weinberg equilibrium.

Logistic regression analysis was used to analyze the genotype allele controlling for age, gender and occupation as the covariables. The results of genotypic and allelic frequency analysis are shown in Table 3. With respect to the frequencies of rs7517847, AR patients and exhibited a significant exhibited a significantdifference. The frequencies of the rs7517847 GG genotype and the minor allele G in the AR patients were very high, unlike that observed in the case of the control individuals (P<0.001, odds ratio (OR) 4.248, 95% CI 2.60 to 6.95; P<0.001, OR 1.62, 95% CI 1.32 to 1.98, respectively). The results also show that the frequencies of rs17375018 and rs1343151 were different between the AR patients and the control individuals, although the significances are only marginal. (P = 0.002, OR 0.48, 95% CI 0.26 to 0.88; P = 0.046, OR 0.51, 95% CI 0.26 to 0.99, respectively). No difference in the genotype frequencies of the rs11209032 SNP was observed between the AR cohort and the control group.

**Table 3:** Frequencies of alleles and genotypes of IL-23R polymorphisms in AR patients and control individuals.

SNP	Genotype	AR (%)	Control (%)	χ2	P	Adjusted	Unadjusted
	Allele	(N = 358)	(N = 407)			OR(95%CI)	OR(95%CI)
rs17375018	GG	176(49.2)	183(45.0)			reference	reference
	AG	150(41.9)	188(46.2)	13.27	0.002	0.50(0.34–0.72)	0.83(0.62–1.12)
	AA	32(8.9)	36(8.8)	5.77	0.02	0.48(0.26–0.88)	0.93(0.55–1.55)
	G	502(70.1)	554(68.1)			reference	
	A	214(29.9)	260(31.9)	0.75	0.39	0.91(0.73–1.13)	
rs7517847	TT	90(25.1)	148(36.8)			reference	reference
	GT	169(47.2)	191(47.5)	16.07	6.11×10^−5^	2.31(1.53–3.48)	1.46(1.04–2.03)
	GG	99(27.7)	63(15.7)	33.12	8.65×10^−9^	4.25(2.60–6.95)	2.58(1.71–3.90)
	T	349(48.7)	487(60.6)			reference	
	G	367(51.3)	317(39.4)	21.41	3.70×10^−6^	1.62(1.32–1.98)	
rs1343151	CC	344(96.1)	377(92.6)			reference	reference
	TC	14(3.9)	30(7.4)	3.97	0.05	0.51(0.26–0.99)	0.51(0.27–0.98)
	TT	0	0	–	–	–	–
	C	702(98.0)	784(96.3)			reference	
	T	14(2.0)	30(3.7)	4.08	0.04	0.52(0.28–0.99)	
rs11209032	AA	66(18.4)	88(21.6)			reference	reference
	AG	198(55.3)	216(53.1)	1.22	0.27	1.24(0.85–1.80)	1.22(0.84–1.78)
	GG	94(26.3)	103(25.3)	0.89	0.35	1.23(0.80–1.88)	1.22(0.80–1.86)
	A	330(46.1)	392(48.2)			reference	
	G	386(53.9)	422(51.8)	0.65	0.42	1.09(0.89–1.33)	

AR, allergic rhinitis; OR, odds ratio; SNP, single-nucleotide polymorphism.

Logistic regression analysis was used to analyze the genotype allele controlling for age, gender and occupation as the covariables.

The Haploview V3.32 program and the online software platform SHEsis were used to analyze the haplotype data and probabilities in Table 4. We used the non-risk haplotype as the reference and tested all other haplotypes. The results show that the AGTG haplotype frequency was significantly low in the AR patients, unlike that observed in the control individuals (P<0.001, OR 0.10, 95% CI 0.023 to 0.43).In addition, a substantially high frequency of the GGCA and GGCG haplotypes was observed in the AR patients, unlike that observed in the control individuals (P<0.001, OR 5.77, 95% CI 3.30 to 10.09; P = 0.011, OR 1.66, 95% CI 1.13 to 2.45, respectively).No difference was detected between the AR patients and the control individuals with respect the other 5 haplotypes (Table 4).

**Table 4:** Frequencies of the haplotypes formed by rs17375018, rs7517847, rs1343151 and rs11209032 SNPs in AR patients and healthy control individuals.

Haplotype	AR (%)	Control(%)	χ^2^	P	Odds Ratio (95%CI)
G T C G	105.22(14.7)	142.18(17.7)			reference
A G T G	2.00(0.3)	27.19(3.4)	13.866	1.96×10^−4^	0. 100(0.023–0.431)
A G C A	21.78(3.0)	35.04(4.4)	0.292	0.589	0. 850 (0.471–1.533)
A G C G	162.26(22.7)	157.32(19.6)	3.824	0.051	1. 395 (0.999–1.950)
A T C A	11.93(1.7)	29.03(3.6)	2.556	0.110	0.560 (0.273–1.148)
G G C A	80.73(11.3)	18.51(2.3)	42.401	7.433×10^−11^	5. 765 (3.294–10.090)
G G C G	96.60(13.5)	78.94(9.8)	6.543	0.011	1. 661 (1.125–2.452)
G T C A	208.90(29.2)	304.37(37.9)	0.215	0.643	0. 930(0.684–1.265)

AR, allergic rhinitis; OR, odds ratio.

We further investigate whether the IL-23R SNPs were associated with certain allergen category of AR, such as house dust mite, pollens and other mixed allergens. The analysis failed to find any association of these parameters with the tested IL-23R SNPs (P>0.05).

## Discussion

This study determined an association between IL-23R polymorphisms and AR susceptibility in a Chinese Han population and confirmed a novel SNP, rs7517847, in IL-23R that has a relationship with AR. The haplotype AGTG was found to protect against AR, whereas other haplotypes, GGCA and GGCG, were associated with susceptibility to AR.

In order to establish a correlation with immune diseases, we have investigated many SNPs. The most common IL-23R SNP related to immune diseases are rs7517847, rs11209032, and rs1343151, although rs17375018 was not shown to be associated with other diseases, it was found to be associated with Behcet’s in our laboratory of Ophthalmology; therefore, these 4 were analyzed in our study. Considering that results on the association of gene polymorphisms with disease can be influenced by many factors, we aimed to ensure the correctness of our results. First, we selected AR patients strictly according to the criteria of ARIA and excluded the ones who did not fit the criteria. Second, we chose unrelated healthy individuals from the same geographic region as that of the AR patients’; all the study participants were similar with respect to their age, sex, occupation, and ethnicity. Finally, to verify the results of genotyping by PCR-RFLP, we repeated the sequencing of 20% of the samples, for which we obtained the exact same results as those obtained for the first genotyping. These approaches were taken to ensure the accuracy of the results of this study.

In previous reports, the strategy of selecting SNPs has been noted in association studies of certain genes with disease. As far as we know, we have for the first time investigated the association of 4 SNPs with allergic disease, and found that only rs7517847 was associated with AR. As stated in our study, the frequency of presence of the GG genotype and G allele of rs7517847 in AR patients (P<0.001, odds ratio (OR) 4.248, 95% CI 2.60 to 6.95; Pc<0.001, OR 1.62, 95% CI 1.32 to 1.98, ) presented a significant increase, suggesting an important susceptibility factor to this disease. These results conform to those of the studies on Crohn’s disease and ulcerative colitis, in which rs7517847 had the strongest association [16], [19]–[21].

Further studies are needed to investigate how rs7517847 is directly involved in susceptibility to AR. The intronic polymorphism rs7517847 might exert its influence by regulating the specific splicing of the gene [22]. The rs7517847 T→G change may influence mRNA length and protein product formation [23]. Encoding for specific subunits of the IL-23 receptor, IL-23R, is mainly reflected on activated/memory T cells and natural killer cells [24]. It is more important that various immune functions of IL-23R bring influence through interaction with its ligand. As disclosed in a recent study, IL-23 is not required for the generation of Th17 cells, but is instead necessary for their maintenance. In humans, interleukin-23 synergizes with interleukin-6 and interleukin-1 to promote Th17 development [25], [26]. The hallmark cytokines produced by human Th17 cells are IL-17A and IL-17F, which are engaged in the recruitment, activation, and migration of neutrophils, as well as IL-22. Th17 cells are potent inducers of tissue inflammation. In CD4+Th17 cells, the interleukin-23/interleukin-17 axis is likely engaged in the clearance of pathogens not sufficiently treated by Th1 or Th2 cells alone, especially at epithelial/mucosal barriers. By inducing the production of IL-6, IL-8, and TNFα, an inflammatory response was promoted and, through IL-23, amplification and stability of the Th17 lymphocytes were regulated, as demonstrated in this study [27]. With the discovery of the Th1 and Th2 immune responses, an increasing number of autoimmune disorders have been found to be associated with aberrant Th17 responses and the IL-23/IL-17 axis [28].Further studies are needed to validate the function of rs7517847.

The association of AR with 3 other SNPs of the IL-23R gene (rs11209032, rs17375018, and rs1343151) has been under investigation. The results show that there are small differences between the AR patients and the control individuals concerning the frequencies of rs17375018 and rs1343151. It is interesting to note that the frequency of the rs17375018 G allele was as high as 70.1% and 68.1% in the AR patients and control individuals, respectively, and the frequency of the rs1343151 T allele was as low as 2.0% and 3.7% in the AR patients and control individuals, respectively. Further studies on the influence of rs17375018 and rs1343151 IL-23R variants on AR will require genetic analysis of a very large cohort. The results of a new UK case–control study and meta-analysis of published series show a significant association between rs11209032 and both AS and IBD [20]; we failed to find any association of this SNP with AR.

Haplotype analysis revealed that the haplotype AGTG conferred a reduced risk of AR, whereas 2 other haplotypes GGCA and GGCG were associated with susceptibility to AR. By analyzing the results presented in Table 3 and 4, it is possible that the haplotype AGTG, which provides protection to AR results from the rs17375018 A and rs1343151 T allele. These results suggest that the T allele of rs1343151 may be protective against this disease, and might possibly play a role in influencing the G allele of rs7517847. The haplotypes GGCA and GGCG affect AR differently. By analyzing our results, it is clear that the GG haplotype formed by the rs17375018 and rs7517847 SNPs is a susceptible haplotype, whereas the AT haplotype formed by these 2 SNPs is a protective haplotype. These results suggest that the rs17375018 and rs7517847 SNPs may play a role in both the processes. Further studies, including gene-gene interactions in AR cohorts, are needed to clarify these results.

As with other studies regarding the association of gene polymorphisms in AR, our study also has limitations. As AR is a heterogenetic disease, the environment is critical for the disease; further studies including gene-gene and gene-environment interactions in AR are needed to clarify the impact of IL-23R on allergic disease. From another point of view, this study only investigated AR patients from a Chinese Han population. However, by performing the test on multiethnic populations, the association of IL-23R with AR found in this study could be clarified. In addition, GWAS coverage was insufficient for many asthma candidate genes, imputation based on these data is reliable but incomplete [29]. Moreover, no study reports a genetic association between IL-23R and allergy/atopy from a GWAS approach. In this sense, we need to conduct a more efficient combination for GWAS.

In conclusion, a strong association between rs7517847 in the IL-23R gene and AR was identified in our study. The GG genotype and G allele of rs7517847 were confirmed as incentive factors to AR. In addition, the biological function of rs7517847, a strongly associated SNP expressed in our study, needs to be further researched. Moreover, we also believe that further studies are required to determine whether rs17375018, rs1343151and haplotype AGTG influence AR. Validation and understanding of this IL-23R relationship and the influence of IL-23R variants on the IL-17A/IL-23 pathway will be important to increase the understanding about the pathogenesis of AR.
